# DeSignate: detecting signature characters in gene sequence alignments for taxon diagnoses

**DOI:** 10.1186/s12859-020-3498-6

**Published:** 2020-04-20

**Authors:** Thomas Hütter, Maximilian H Ganser, Manuel Kocher, Merima Halkic, Sabine Agatha, Nikolaus Augsten

**Affiliations:** 1 Department of Computer Sciences, University of Salzburg, Jakob-Haringer-Straße 28, Salzburg, 5020 Austria; 2 Department of Biosciences, University of Salzburg, Hellbrunnerstraße 34, Salzburg, 5020 Austria

**Keywords:** Phylogenetics, Taxonomy, Bioinformatics, Algorithms

## Abstract

**Background:**

Molecular characters have been added in integrative taxonomic approaches in recent years. Nevertheless, taxon diagnoses are still widely restricted to morphological characters. The inclusion of molecular characters into taxon diagnoses is not only hampered by problems, such as their definition and the designation of their positions in a reference alignment, but also by the technical effort.

**Results:**

*DeSignate* is a tool for character-based taxon diagnoses that includes a novel ranking scheme. It detects and classifies individual and combined signature characters (diagnostic molecular characters) based on so-called character state vectors. An intuitive web application guides the user through the analysis process and provides the results at a glance. Further, formal definitions and a uniform terminology of characters are introduced.

**Conclusions:**

*DeSignate* facilitates the inclusion of diagnostic molecular characters and their positions to complement taxon diagnoses. Compared to previous solutions, the tool simplifies the workflow and improves reproducibility and traceability of the results. The tool is freely available as a web application at (https://designate.dbresearch.uni-salzburg.at/) and is open source (https://github.com/DatabaseGroup/DeSignate/).

## Background

Historically, taxonomic diagnoses are restricted to morphological characters distinguishing a particular taxon (the query group) from related taxa (the reference group). Best practice for taxonomic studies suggests an integrative approach combining morphological, molecular, ecological, and physiological data [1–3]. Previous suggestions for applying divergence cut-off values of gene sequences to discriminate and define taxa (threshold-based approach), however, are based on the overall dissimilarity and are not character-based, i.e., do not use distinct molecular characters for separation and characterization [1, 4]. In the character-based approach, each position of an alignment represents a molecular character which may adopt different states in gene sequence data (e.g., nucleotides and deletions). Diagnostic molecular characters are included in taxon diagnoses (e.g., of protists [5] or animals [1, 6–8]). However, data from (potentially) related taxa for comparison with the type species are often lacking or difficult to obtain [9]. Furthermore, available data is frequently not added consistently to formal diagnoses [10, 11], due to problems in, for instance, the definition of diagnostic molecular characters and the designation of their positions, as well as the lack of suitable tools. For a standardized designation of the position of diagnostic molecular characters in taxon diagnoses, a reference sequence alignment and/or a reference sequence are recommended, facilitating comparability and reproducibility [1].

Current software solutions that identify diagnostic molecular characters and their positions suffer from various limitations: The workbench utility in BOLD [12] restricts the selection of query groups to given taxonomical ranks; the analysis with CAOS [13] involves a complex workflow and was developed for classifying unidentified gene sequences. The output data of both tools must be further processed to interpret the results for taxon diagnoses.

Simultaneously with the development of our tool, the R-package QUIDDICH was established by Kühn and Haase [14], emphasizing the need for a tool that detects diagnostic molecular characters to complement formal taxon descriptions, especially for morphologically cryptic taxa. QUIDDICH considers four different types of characters, which partially allows variability within the query group and a restricted comparison to only certain members of the reference group.

In this work, a different but complementing strategy is considered, which focuses on characters that have a uniform character state in the query group. For diagnostic purposes, only those characters that unequivocally discriminate the query group from the reference group are used.

Here, *DeSignate*, an innovative tool for detecting such diagnostic characters and their positions for taxon diagnoses, is introduced together with a formally defined terminology. The analysis is based on a novel representation of the gene sequence data, which enables a ranking of all alignment positions according to their diagnostic relevance, and subsequently their classification. The ranking enables the user to inspect and evaluate all positions. Furthermore, *DeSignate* detects diagnostic combinations of characters. Taking up the suggestion of Jörger and Schrödl [15], the tool provides a graphical web interface that guides the user step-by-step through the analysis and presents the results without need to post-process the output data.

## Implementation

### Character state vectors

*DeSignate* introduces so-called *character state vectors* to achieve a clear and compact representation of the alignment data. Each alignment position within the query and reference groups is represented as an *n*-dimensional vector, where *n* is the number of different character states, i.e., nucleotides, gap (deletion), and missing information. Intuitively, a vector holds a counter for each character state.

#### **Example 1**

**Fig. 1:**
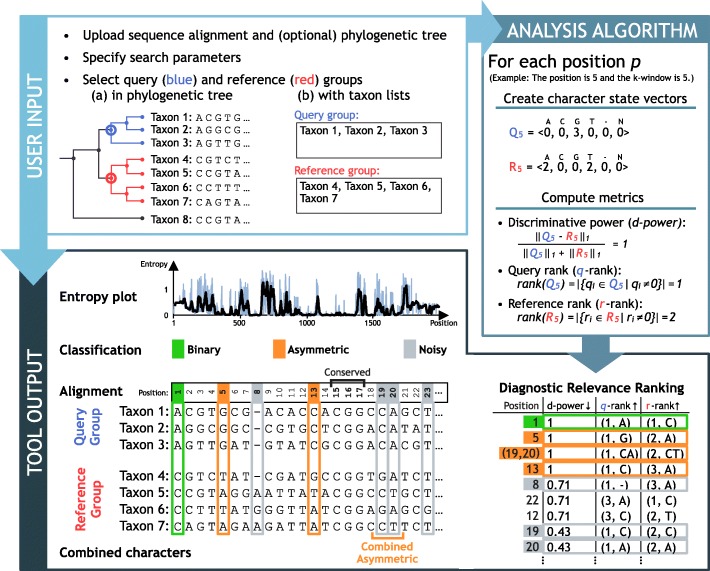
Workflow of *DeSignate*. The data analysis commences with the selection of input data, viz., a sequence alignment and (optionally) the corresponding phylogenetic tree. Next, the query and reference groups are selected in the tree or via comma-separated lists, and the search parameters (e.g., size of *k*-window) are specified. The algorithm ranks all positions in the alignment and identifies both individual and combined candidate characters based on the discriminative power, the query rank, and the reference rank of the respective character state vectors. As proposed by Hadziavdic et al. [16], the entropy plot displays variable (peaks) and conserved regions in the alignment

Given the query group *Q* and the reference group *R* of the taxa in Fig. 1 and a list $\mathcal {B} = \langle A, C, G, T, -, N \rangle $ of possible character states. The resulting $|\mathcal {B}|$-dimensional character state vectors for position *p*=5 are *Q*_5_=〈0,0,3,0,0,0〉 and *R*_5_=〈2,0,0,2,0,0〉.

### Diagnostic relevance ranking

All alignment positions *p* of a query group with respect to a reference group are ranked based on three metrics. (1) The *discriminative power* (d-power) is defined as the normalized *L*_1_-distance, denoted $\frac {||Q_{p} - R_{p}||_{1}}{||Q_{p}||_{1} + ||R_{p}||_{1}}$, of the character state vectors. If the query and reference groups have no character state in common at position *p*, the discriminative power takes the maximum value of 1. (2) The *query rank* (q-rank) is the number of non-zero dimensions of the query character state vector, denoted *rank*(*Q*_*p*_)=|{*q*_*i*_∈*Q*_*p*_∣*q*_*i*_≠0}|. Similar to the query rank, (3) the *reference rank* (r-rank) is the number of non-zero dimensions of the reference character state vector. The *diagnostic relevance ranking* lexicographically orders all positions by discriminative power (descending), query rank (ascending), and reference rank (ascending). Hence, the most relevant positions for taxon diagnoses are listed first.

The diagnostic relevance ranking in Fig. 1 further displays either the uniform or the prevalent character state at each position next to the query and reference ranks.

### Candidate character classification

Based on the discriminative power, the query rank, and the reference rank of character state vectors, a consistent terminology is introduced, and classes of candidate characters are formally defined.

For the implementation into taxon diagnoses only homologous alignment positions of the query group with an identical character state, named candidate characters, are suitable (cf. Definition 1). Note that positions with missing information in the query group (character state N) are not considered candidate characters.

#### **Definition 1**

(Candidate character) Given a query group *Q*, position *p* is a *candidate character* if and only if the query rank is 1, *rank*(*Q*_*p*_)=1, and the dimension for missing information is 0, $Q_{p_{N}} = 0$.

Candidate characters are further classified as (1) *conserved*: uniform and consistent in both the query and the reference groups, (2) *noisy*: at least one character state in the reference group is identical to the character state in the query group, (3) *asymmetric*: the character states of the reference group are not uniform but different from the character state in the query group, or (4) *binary*: the character states of the reference group are uniform but different from the character state in the query group. The categories binary, asymmetric, and noisy follow the suggestions of Wägele and Rödding [17].

#### **Example 2**

Figure 1 shows ten classified candidate characters; position 1 is binary, positions 5 and 13 are asymmetric, positions 8, 19, 20, and 23 are noisy, and positions 15, 16, and 17 are conserved.

In taxon diagnoses, only characters that distinguish all members of the query group from all members of the reference group, so-called signature characters (cf. Definition 2), are of interest. Based on the classification scheme in Table 1, only binary and asymmetric candidate characters shall thus be considered.

**Table 1:** Classification of candidate characters

Vector metrics				
Classes	d-power	q-rank	r-rank	
Binary	1	1	1	$\left.\begin {array}{l} \\ \\ \end {array}\right \rbrace { \parbox {0.5cm}{signature \\ characters}}$
Asymmetric	1	1	>1	
Noisy	<1	1	>1	
Conserved	<1	1	1	

#### **Definition 2**

(Signature character) A candidate character at position *p* is a *signature character* if and only if its discriminative power is 1.

### Combined characters

Typically, many candidate characters are classified as noisy and are therefore unsuitable for unambiguously distinguishing the query from the reference group. However, individual noisy candidate characters may pair to asymmetric combinations with a discriminative power of 1. To reduce the number of analyzed combinations, a so-called *k-window* is introduced. Given an integer value *k*≥1 and two noisy candidate characters at positions *i* and *j*, a *combined character* (*i*,*j*) is considered if |*i*−*j*|<*k*. Since the combination of two candidate characters results in character pairs (e.g., TT, TC, GA), the character state vectors increase in size ($|\mathcal {B}|^{2}$ rather than $|\mathcal {B}|$ dimensions).

#### **Example 3**

Consider the noisy candidate characters at positions 8, 19, 20, and 23 in Fig. 1 and a *k*-window of size *k*=5. Due to the *k*-window, only the combined characters (19,20), (19,23), and (20,23) are considered. The combined characters (19,23) and (20,23) are noisy (CT respectively AT occur in both the query and the reference groups); however, (19,20) is asymmetric since its discriminative power is 1, the query rank is 1 (only CA), and the reference rank is 2 (GA and CT).

Example 3 considers the combination of only two candidate characters. In general, the technique to combine candidate characters and rank their character state vectors is extensible to any number of positions.

## Results and discussion

The presented tool *DeSignate* detects and classifies individual and combined candidate characters in a gene sequence alignment based on novel methods outlined in section “Implementation”.

### Key results and features

The key results and the features of *DeSignate* include:

- **Usability:** In the web interface of *DeSignate*, the user is guided through the analysis process in simple steps as illustrated in Fig. 1: (1) the input data are uploaded by the user in the form of a gene sequence alignment and (optionally) the corresponding phylogenetic tree, (2) the search parameters (e.g., *k*-window) are specified, and (3) the query and reference groups are defined by selecting subtrees of the input tree. Alternatively, the query and reference groups are defined by comma-separated taxon lists, if no tree is uploaded. The current version of the tool supports the combination of up to two candidate characters.
- **Visualization and interpretation of the results:** The results are displayed after ranking and classifying all alignment positions, i.e., all molecular characters. The output (cf. Fig. 1) includes (1) a *signature bar* showing all binary, asymmetric, and noisy candidate characters and their positions in the sequence alignment, (2) an *entropy plot* indicating the variable sequence regions by peaks based on the input alignment, (3) an alignment of the query and reference groups with color-coded binary, asymmetric, and noisy candidate characters, and (4) a table of all molecular characters ordered according to the *diagnostic relevance ranking* introduced above.
- **Free availability:** The software of *DeSignate* is implemented in Python 3 using Django, and the source code is publicly available on Github at https://github.com/DatabaseGroup/DeSignate/. Experienced users can execute the tool using the command-line; additionally, a user-friendly and intuitive web application is provided at https://designate.dbresearch.uni-salzburg.at/.
- **Uniform terminology:** A uniform terminology is defined based on the introduced metrics of character state vectors, which further facilitates the inclusion of signature characters in taxon diagnoses.
- **Further applications:** Next to taxon diagnoses, envisaged applications comprise the support of designing group-specific primers to target certain taxa in natural communities for better diversity estimates [18] and the relation of unknown molecular diversity to morphotypes by using FISH probes [19].
  The flourishing field of environmental sequencing generates an enormous amount of data. In ecological and biogeographical analyses, the gene sequences found in the samples are compared with a reference databank. Using a certain threshold similarity, the new sequences are annotated with the most closely related taxon’s name. This annotation step can distinctly be improved by a character-based approach using the molecular characters included in the taxon diagnoses. Even if no identical or highly similar sequence can be found in the database, the signature characters detected by *DeSignate* enable a more precise taxon assignment/classification and thus increase the taxonomic resolution of the analyses. As a result, the inference of the organisms’ pecularities from the literature are more precise, allowing more reliable estimates of ecological processes.

### Comparison with existing solutions

Currently, time-consuming inspections of the alignments by eye yield characters distinguishing the query group from the reference group, e.g., by Agatha and Strüder-Kypke [10]. Previous software solutions like the workbench utility in BOLD [12] and CAOS [13] are rarely employed. In contrast to these tools, *DeSignate* combines easy accessibility with flexible analysis capabilities and a comprehensive but clear overview of the results. The major differences (cf. Table 2) comprise (1) the availability of a web and a command-line interface, (2) the supply of an open source license, (3) the support of standard sequence alignment and tree file formats (CAOS requires manual editing of the input files; BOLD requires data to be curated into its database), (4) the free choice of the query and reference groups based on the selection in a tree or via comma-separated lists (CAOS requires a new tree for each comparison; BOLD is restricted to taxonomic ranks predefined in its database), (5) the analysis of combined nucleotides within a user-defined window (CAOS also supports the analysis of two position combinations, but details on the underlying algorithm are missing), (6) a clear representation of the results that can readily be verified without further processing of the data (CAOS requires the extraction of diagnostic characters from the output file; BOLD only provides a list of diagnostic characters).

**Table 2:** Comparison of features for detecting diagnostic characters between *DeSignate*, BOLD’s Diagnostic Characters workbench utility [12], CAOS [13], and QUIDDICH [14]

Features	*DeSignate*	BOLD	CAOS	QUIDDICH
Web interface	+	+	−	−
Command-line interface	+	−	+	+
Open source license	+	−	−	+
Requires no editing of input files	+	+	−	+
Free choice of nucleotide sequence alignment	+	+	+	+
Group selection with a (guide) tree	+	−	+	−
Free choice of the query and reference groups	+	−	+	+
Analysis of combined characters	+	−	+	−
Support for interpretation and validation	+	−	−	−

The recently published R package QUIDDICH developed by Kühn and Haase [14] markedly differs from *DeSignate* in its definitions of diagnostic molecular characters. QUIDDICH is more relaxed regarding the variability within the query group by allowing different character states (type 2 characters) and by allowing the detection of character states only distinguishing some members of the query group from the reference group (type 3 characters).

In contrast, *DeSignate* is more stringent by only considering uniform character states (candidate characters) in the query group for the comparison with the reference group. Furthermore, binary and asymmetric characters (signature characters) detected by *DeSignate* are subsumed under one definition in QUIDDICH (type 1 characters). Additionally, QUIDDICH extracts pairwise diagnostic characters for the query group compared to at least one taxon in the reference group; both have a uniform but different state (type 4 character) corresponding to the definition of binary characters in *DeSignate*.

QUIDDICH does not feature a web-interface but allows an efficient workflow for users familiar with the R environment. The output consists of a table compiling the classified alignment positions (= molecular characters) with their type, character states, and the compared taxa; however, the tool does not include features for the interpretation and validation of the output.

For comparing QUIDDICH and *DeSignate* [see Additional file 1], an analysis with the dataset (nucleotide sequence alignment of 51 species) from the electronic supplementary material of Kühn and Haase [14] was conducted. The same four taxa (represented by up to eleven sequences each) were chosen in separate analyses as query groups. *DeSignate* detected all diagnostic characters defined as type 1 (binary + asymmetric) and type 4 (pairwise binary) in QUIDDICH. Type 2 and type 3 characters determined by QUIDDICH were not classified by *DeSignate*, as they are not conform with the definition of candidate characters, i.e., identical character states in the query group. Nevertheless, the characters are highly rated in the diagnostic relevance ranking owing to the discriminative power of their vector metrics [see Additional file 1, Table 2].

The capability to analyze combined characters by *DeSignate* yielded 31, 104, 521, and 628 diagnostic character combinations for the four taxa, respectively, providing a high potential discriminative power of this method. The current version of QUIDDICH does not analyze combined characters.

In sum, QUIDDICH and *DeSignate* differ in their requirements concerning uniformity of the character states in the query group (relaxed vs. stringent) and thus partially deviate in their fields of application.

## Conclusion

*DeSignate* is a novel tool for detecting signature characters to complement taxon diagnoses. Compared to previous solutions, *DeSignate* provides a user-friendly and rapid workflow, enables reproducibility and traceability of the results, introduces a uniform and well-defined terminology, and includes the analysis of combined characters.

## Supplementary information

**Additional file 1** XLSX file containing summarized and detailed results of the comparison between QUIDDICH and *DeSignate*.
